# A *Plasmodium falciparum* copper-binding membrane protein with copper transport motifs

**DOI:** 10.1186/1475-2875-11-397

**Published:** 2012-11-29

**Authors:** David L Choveaux, Jude M Przyborski, JP Dean Goldring

**Affiliations:** 1 Biochemistry, University of KwaZulu-Natal, P.B. X01, Carbis Road, Scottsville, 3209, South Africa; 2 Parasitology, Faculty of Biology, Philipps University Marburg, Karl von Frisch Str. 8, Marburg, D-35043, Germany

**Keywords:** Malaria, Copper transporter, Permeome, PEXEL-negative

## Abstract

**Background:**

Copper is an essential catalytic co-factor for metabolically important cellular enzymes, such as cytochrome-c oxidase. Eukaryotic cells acquire copper through a copper transport protein and distribute intracellular copper using molecular chaperones. The copper chelator, neocuproine, inhibits *Plasmodium falciparum* ring-to-trophozoite transition *in vitro*, indicating a copper requirement for malaria parasite development. How the malaria parasite acquires or secretes copper still remains to be fully elucidated.

**Methods:**

PlasmoDB was searched for sequences corresponding to candidate *P. falciparum* copper-requiring proteins. The amino terminal domain of a putative *P. falciparum* copper transport protein was cloned and expressed as a maltose binding fusion protein. The copper binding ability of this protein was examined. Copper transport protein-specific anti-peptide antibodies were generated in chickens and used to establish native protein localization in *P. falciparum* parasites by immunofluorescence microscopy.

**Results:**

Six *P. falciparum* copper-requiring protein orthologs and a candidate *P. falciparum* copper transport protein (PF14_0369), containing characteristic copper transport protein features, were identified in PlasmoDB. The recombinant amino terminal domain of the transport protein bound reduced copper *in vitro* and within *Escherichia coli* cells during recombinant expression. Immunolocalization studies tracked the copper binding protein translocating from the erythrocyte plasma membrane in early ring stage to a parasite membrane as the parasites developed to schizonts. The protein appears to be a PEXEL-negative membrane protein.

**Conclusion:**

*Plasmodium falciparum* parasites express a native protein with copper transporter characteristics that binds copper *in vitro*. Localization of the protein to the erythrocyte and parasite plasma membranes could provide a mechanism for the delivery of novel anti-malarial compounds.

## Background

Malaria is a serious, acute and chronic relapsing infection that kills close to one million people annually. More than 90% of these deaths are recorded in the sub-Saharan regions of Africa, with the majority being children under the age of five years [1]. Human mortality, as a result of malaria infection, is predominantly caused by *Plasmodium falciparum*. Efforts to prevent and control the disease have, however, been hindered by an increased parasite resistance to currently available anti-malarial drugs, highlighting the need for novel anti-malarial drug development [2]. A group of proteins gaining increased interest as potential therapeutic targets are the integral membrane proteins predicted to possess transport functions [3,4]. Despite these proteins playing key roles in *Plasmodium* parasite growth and replication [4], they remain poorly understood and underexploited [3]. Previous studies employing the intracellular copper chelator neocuproine established that copper is an essential micronutrient for *in vitro* parasite growth [5]. Rasoloson *et al.*[5] described the membrane-bound *Pf*CuP-ATPase copper efflux protein and suggested this protein acts to reduce copper toxicity in *P. falciparum*. Studying *P. falciparum* copper metabolism may lead to the identification of novel anti-malarial drug targets.

Copper is an essential micronutrient that plays important catalytic and structural roles in numerous enzymes. Cuproenzymes harness the ability of copper to cycle between a stable oxidized Cu(II) and unstable reduced Cu(I) state for various redox reactions. However, this property also makes copper potentially toxic to cells since it can undergo free radical producing Fenton chemistry [6]. Consequently, cells have evolved homeostatic mechanisms for the uptake, distribution, sequestration and secretion of copper to meet essential cellular requirements while reducing its toxic potential. From yeast to humans, copper acquisition is mediated by the high affinity copper transport protein, Ctr1 [7,8]. Copper metallochaperones subsequently distribute intracellular copper to specific proteins or organelles. The copper chaperone for superoxide dismutase (CCS) distributes copper to cuprozinc superoxide dismutase (Cu/Zn SOD) in the cytosol and mitochondrion, antioxidant protein 1 (Atox1) transfers copper to the secretory pathway and nucleus and an ensemble of proteins deliver copper to cytochrome-c oxidase (CCO) in the mitochondrion [7,8]. It has been suggested that Cu-ATPase-mediated secretion of excess copper is the main factor regulating copper homeostasis [9]. Equally important to mammalian growth and development, however, is the copper transporter Ctr1. Cell-specific knock-out of *Ctr1* in mouse intestinal epithelial cells caused marked growth retardation, coupled with cardiac hypertrophy and overall viability defects that caused postnatal lethality around three weeks of age [10]. Similarly, knock-out of the *Drosophila melanogaster Ctr1A* gene resulted in developmental arrest at early larval stages [11]. The C terminus of yeast copper transport protein, Ctr1, appears to have a role in copper regulation and stopping the build-up of copper to toxic concentrations [12].

Although a *P. falciparum* P-type ATPase copper efflux protein has been described [5], an additional protein-mediated mechanism for copper acquisition has not. The current study describes a novel membrane-bound *P. falciparum* protein with copper transporter characteristics. A recombinant form of the protein's amino terminal region bound reduced copper *in vitro* and within *E. coli* host cells. The native *P. falciparum* copper transport protein was found to initially localize to the infected erythrocyte membrane, followed by relocation to a parasite membrane through the development of the parasite’s erythrocytic cycle.

## Methods

### Bioinformatics

The annotated *Plasmodium* database [13] was screened for the presence of copper-requiring protein orthologs using a BlastP search. Sequences used to screen the database are listed in Table 1. A putative *Plasmodium* copper transport protein was identified using the *Theileria parva* (Muguga stock) polymorphic immunodominant molecule (GenBank:AAA99499) [14] to BlastP screen PlasmoDB. In an effort to support sequence identity, the putative *Plasmodium* copper transport protein sequences were aligned with characterized copper transporter sequences from *Homo sapiens* (NCBI:NP_001850), *Arabidopsis thaliana* (Genbank:BAE98928) and *Saccharomyces cerevisiae* (Genbank:AAB68064) using the ClustalW™ server. Transmembrane domains were identified in the putative *Plasmodium* copper transporters using the HMMTOP [15] and TMHMM 2.0 [16] topology prediction servers. The presence of potential signal sequences in each protein was established using the TMHMM server and the SignalP 3.0 program (Center for Biological Sequence Analysis, Lyngby, Denmark). A cladogram was generated from a ClustalW™ alignment of six putative *Plasmodium* copper transporters with seven characterized copper transporters. The characterized transporters were from *Homo sapiens*, *Mus musculus* (NCBI:NP_780299), *Rattus norvegicus* (NCBI:NP_598284), *Danio rerio* (NCBI:NP_991280), *Arabidopsis thaliana*, *Saccharomyces cerevisiae* and *Theileria parva*.

**Table 1:** **Copper-dependent protein orthologs to *Homo sapiens *proteins identified in the *P. falciparum *proteome**

**Enzyme name**	**Accession number**	***P. falciparum *ortholog identified**	**PlasmoDB protein identifier**
CCS	CAG46726	No	N/A
Cu/Zn Superoxide dismutase	AAB05661	No	N/A
Dopamine-β-monooxygenase	PO9172	No	N/A
Peptidylglycine monooxygenase	P19021	No	N/A
Metallothionein	AAP97267	No	N/A
S-adenosyl-L-homocysteine hydrolase	P23526	Yes	PFE1050w
Cox11	CAG46636	Yes	PF14_0721
Cox17	AAA98114	Yes	PF10_0252
Cox19	AAY35062	Yes	PFL0090c
Cg3	O75880	Yes	PF07_0034
Cytochrome-c oxidase subunit I	ACR77861	Yes	mal_mito_2
Cytochrome-c oxidase subunit II	ABU47824	Yes	PF14_0288 (IIa) PF13_0327 (IIb)
Cytochrome-c oxidase subunit III	ABJ99455	Yes	mal_mito_1
Cytochrome-c oxidase subunit Vb	AAA52060	Yes	PFI1365w
Cytochrome-c oxidase subunit VIb	AAP35591	Yes	PFI1375w

### Antibody production and purification

The amino acid sequence for a putative *P. falciparum* copper transport protein (PF14_0369) was retrieved from PlasmoDB and subjected to Predict7 [17] analysis to identify immunogenic regions and the peptide CNLQKEEDTVVQLQD was selected for synthesis (GL Biochem Ltd. Shanghai, China). The selected peptide was coupled to rabbit albumin via the native amino terminal cysteine residue using m-maleimidobenzoyl-N-hydroxysuccinimide ester. Hy-Line Brown hens were used for antibody generation and antibodies were isolated from egg yolk as previously described [18,19]. Animal ethics clearance was obtained from the University of KwaZulu-Natal animal ethics committee (003/10/Animal).

### PCR amplification and cloning

*Plasmodium falciparum* (D10) gDNA was isolated from infected red blood cells using the Fermentas^TM^ DNA purification kit. The full-length PF14_0369, open reading frame was amplified using the following specific primers: *Pf*Ctr369-fwd: 5'-*at*GAATTCGACAAAAGCGACAATAGTATTTG-3' and *Pf*Ctr369FL-rev: 5'-ACATCCACAACAAGCTGGATC. For functional copper binding studies, the coding domain for the protein's amino terminal domain minus signal peptide was amplified using the *Pf*Ctr369-fwd primer containing an engineered *Eco*RI cleavage site and *Pf*Ctr369Nt-rev: 5'-*ca*CTGCAGTTACGATTTGGTTTCCCATTTG-3' containing a *Pst*I cleavage site (cleavage sites underlined). PCR products were analyzed on a 1% (w/v) agarose gel. The PCR amplicon for the PF14_0369 amino terminal domain (designated Nt) minus signal peptide (*Pf*Ctr369Nt^-S^) was cloned in frame with the maltose binding protein (MBP) gene in the pMal-p2x bacterial expression vector (New England BioLabs, USA), as previously described [18].The integrity of the resultant pMal-p2x/*Pf*Ctr369Nt^-S^ plasmid insert was confirmed by DNA sequencing. Using the strategy employed for pMal-p2x/*Pf*Ctr369Nt^-S^, a plasmid containing pMal-p2X/*Pf*Ctr211Nt^-S^ from the PF14_0211 gene was prepared.

### Expression and purification of recombinant MBP-*Pf*Ctr369Nt^-S^

A single *E. coli* JM103[pMal-p2x/*Pf*Ctr369Nt^-S^ colony was grown overnight at 30°C in sterile LB medium supplemented with 100 μg.ml^-1^ ampicillin. The overnight culture was diluted in fresh broth, plus antibiotic, and incubated at 30°C until the A_600_ reached ~0.6. MBP-*Pf*Ctr369Nt^-S^ expression was induced with 0.3 mM IPTG and the cultures grown for a further 16 h before harvesting of cells by centrifugation (4000 *g*, 15 min, 4°C). The *E. coli* periplasmic content was isolated following the method described by French *et al.*[20] and the recombinant protein purified by amylose affinity chromatography. Recombinant MBP-*Pf*Ctr211Nt^-S^ was expressed using the same procedure. Protein samples were analyzed on a 12.5% SDS-PAGE gel [21] followed by Western blotting and probed with chicken anti-PF14_0369 and anti-MBP antibodies [22] (see below). Protein concentration was determined with the Bradford dye-binding protein assay, using ovalbumin as the standard [23].

### Parasite culture

*Plasmodium falciparum* 3D7 parasites were cultured in human O^+^ erythrocytes according to standard protocols, except cultures were incubated in gassed flasks [24]. Synchronized asexual parasites were obtained by sorbitol treatment [25].

### Western blot analyses

For Western blot analyses of *P. falciparum* lysates, protein samples were prepared from saponin-isolated, sorbitol-synchronized parasites. Equivalent amounts of 1 x 10^7^ parasites were analysed by 12.5% SDS-PAGE and transferred to a nitrocellulose membrane. Primary chicken anti-PF14_0369 and chicken anti-*P. falciparum* lactate dehydrogenase (LDH) antibodies against specific peptides on each protein were employed and detected using rabbit anti-chicken horseradish peroxidase-conjugated secondary antibodies (DAKO, Santa Cruz). Immunoblots were developed using enhanced chemiluminescence. Recombinant MBP-*Pf*Ctr369Nt^-S^ expression was analysed by Western blot using chicken anti-MBP and anti-PF14_0369 anti-peptide antibodies. These antibodies were detected using rabbit anti-chicken horseradish peroxidase-conjugated secondary antibodies (Jackson Immuno-Research Laboratories, USA) and visualized by incubating the membrane in 0.06% (w/v) 4-chloro-1-naphthol and 0.0015% (v/v) H_2_O_2_.

### Copper binding

For *in vitro* copper binding studies, purified MBP-*Pf*Ctr369Nt^-S^ (10 μM) was mixed with 200 μM CuCl_2_, with or without 10 mM ascorbic acid, and incubated at RT for 15 min. Unbound copper was removed by overnight dialysis against sodium phosphate buffer [0.1 M NaH_2_PO_4_, 0.01% (w/v) NaN_3_, pH 7.5] at 4°C. Copper binding to MBP-*Pf*Ctr369Nt^-S^ and the oxidation state of bound copper was assessed using the bicinchoninic acid (BCA) assay [26], in which protein-copper complexes are disrupted by acid denaturation and copper detected upon release. BCA binds reduced copper (Cu^+^), forming a purple complex detectable at 354 nm. If copper is present in solution as Cu^2+^, ascorbate must be added to the sample following acid denaturation to yield a Cu^+^-BCA complex. However, if the addition of ascorbate does not influence Cu^+^-BCA complex formation, this indicates copper was released from the protein in the Cu^+^ state. Solutions of CuCl_2_ in sodium phosphate buffer were used as standards and affinity purified MBP served as a negative control. The ability of MBP-*Pf*Ctr369Nt^-S^ to bind copper within *E. coli* cells was also examined. Cells containing the recombinant protein were grown in the presence or absence of 0.5 mM CuCl_2_, and the recombinant protein purified as described above. Prior to cell disruption and MBP-*Pf*Ctr369Nt^-S^ isolation, pelleted cells were washed with a Tris buffer (0.2 M Tris–HCl, pH 7.5) to remove extraneous CuCl_2_. As with *in vitro* copper binding, copper bound to MBP-*Pf*Ctr369Nt^-S^ within *E. coli* cells was detected using the BCA assay.

### Ascorbate oxidation assay

The ability of MBP-*Pf*Ctr369Nt^-S^ to inhibit the copper-catalysed oxidation of ascorbic acid (H_2_Asc) was tested *in vitro*. In a typical experiment a mixture was prepared containing 5 μM MBP-*Pf*Ctr369Nt^-S,^, or 5 μM MBP (control), 8 μM CuCl_2_ and 120 μM H_2_Asc solution, adjusted to pH 4.5 with NaOH. The absorbance of this solution was monitored at 255 nm with readings taken every 5 s for 300 s. Reactions were carried out at RT.

### Immunofluorescence

Immunofluorescence assays were carried out following cell fixation with 4% paraformaldehyde/0.00075% (w/v) glutaraldehyde as previously described [27] except fixation was carried out at 37°C for 30 minutes and quenching was performed with 125 mM glycine/PBS. Chicken anti-peptide antibodies directed against *P. falciparum* lactate dehydrogenase and PF14_0369 and a rabbit antibody against Exp-1 were used. Primary antibodies were diluted in 3% (w/v) BSA/PBS and detected with either goat anti-chicken-Cy3 or goat anti-rabbit-Cy2 antibodies (DAKO, Santa Cruz). Hoechst 33258 (Molecular probes) was used at a concentration of 50 ng.ml^-1^ to stain parasite DNA. All images were acquired at room temperature on a Zeiss Cell Observer using appropriate filter sets.

## Results

### Identification of copper-dependent protein orthologs in the *P. falciparum* proteome

Cytochrome*-*c oxidase (CCO) is a key multi-subunit catabolic protein containing three functionally important copper ions in its binuclear Cu_A_ and mononuclear Cu_B_ sites [28]. Metallation of the Cu_A_ and Cu_B_ sites requires the copper metallochaperones Cox17, Sco1 and Cox11 [29]. Identification of *P. falciparum* orthologs of these metallochaperones and other related copper-requiring proteins supports a plasmodial requirement for copper, as previously suggested [5]. A BlastP search of PlasmoDB, using mammalian and yeast proteins, identified six copper binding protein orthologs (Table 1). These were Cox11, Cox17, Cox19, Sco1, five CCO subunits and S-adenosyl-L-homocysteine hydrolase. The identification of CCO subunit I and II orthologs appears most important since these house the binuclear Cu_A_ and mononuclear Cu_B_ copper binding sites [28]. Interestingly, the *P. falciparum* proteome appears to lack an identifiable class of metallothioneins and Cu/Zn SOD (Table 1). In yeast and mammalian cells, these proteins are essential for copper storage and free radical detoxification, respectively [7,8].

### Identification of a putative *Plasmodium* copper transport protein

**Figure 1:**
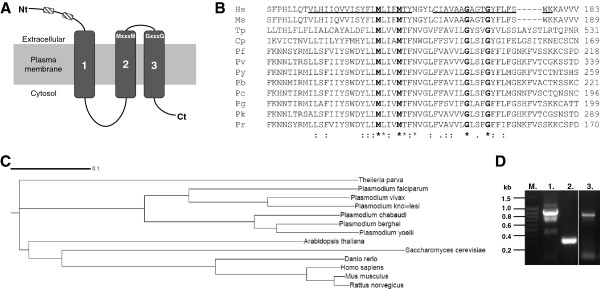
**Features characteristic of the copper transport protein family are conserved in the putative *Plasmodium *copper transporters. A**. Three transmembrane (1–3), an extracellular amino terminal (Nt) and intracellular carboxy terminal (Ct) domains, methionine and MxxxM and GxxxG motifs of copper transport proteins. **B**. An aligned region of the *Plasmodium* and other copper transporters showing the MxxxM and GxxxG motifs. Sequence identifiers are: Hs = *Homo sapiens*; Ms = *Mus musculus*; Tp = *Theileria parva*; Cp = *Cryptosporidium parvum*; Pf = *P. falciparum* PF14_0369; Pv = *P. vivax*; Py = *P. yoelii*; Pb = *P. berghei*; Pc = *P. chabaudi*; Pg = *P. gallinaceum*; Pk = *P. knowlesi*; Pr = *P. reichenowi*. Underlined amino acids (Hs) indicate the positions of transmembrane domains two and three. **C**. Cladogram comparing six complete *Plasmodium* copper transporter sequences with seven characterized transporters from various organisms. **D**. PCR amplification of the PF14_0369 coding domain from *P. falciparum* gDNA. Lane 1, full-length PF14_0369 coding domain; lane 2, amino terminal domain of PF14_0369; lane 3, *P. falciparum* LDH (PF13_0141) control. A Fermentas DNA ladder (M) is shown to the left of the gel.

Although *P. falciparum* appears to require copper for survival [5], the mechanism by which the parasite acquires copper remains to be formally identified. One suggested mechanism is acquisition via the ingestion and digestion of host erythrocyte Cu/Zn SOD [5]. A potential alternate mechanism is acquisition via a copper transport protein. The *Theileria parva* polymorphic immunodominant molecule sequence [14], which possesses copper transport protein features, was used to screen PlasmoDB for a candidate *Plasmodium* copper transport protein. Full length, putative copper transporter sequences were identified for six *Plasmodium* species, namely *Plasmodium falciparum* (PF14_0369 and PF14_0211), *Plasmodium vivax* (PVX_118540), *Plasmodium yoelIi* (PY00413), *Plasmodium berghei* (PBANKA_130290)*, Plasmodium chabaudi* (PCHAS_130610), and *Plasmodium knowlesi* (PKH_126730). Incomplete sequences possessing copper transporter features were identified for *Plasmodium gallinaceum* (Pgal0564e04) and *Plasmodium reichenowi* (reich164f12.q1k). Analysis of the complete *Plasmodium* sequences with HMMTOP [15] and TMHMM [16] identified at least three putative transmembrane domains and a signal peptide in the sequences (Figure 1A). The *Plasmodium* copper transport protein sequences contain characteristic features of the copper transport protein family, including three transmembrane domains, copper-binding methionine motifs within the extracellular amino-terminal domain (Figure 1A) and the essential MxxxM (Mx_3_M) and GxxxG (Gx_3_G) motifs (Figure 1B) in the second and third transmembrane domains respectively. An essential methionine residue, located 20 amino acid residues from the first putative transmembrane domain [30], is present in all the *Plasmodium* sequences (Additional file 1).

A cladogram was generated for the complete *Plasmodium* copper transporters to compare their similarity to seven characterized copper transporters. The *Plasmodium* copper transporters formed a cluster related to, but separate from, the characterized copper transporters (Figure 1C). Of the characterized transporters, the sequence with the greatest similarity to that of *Plasmodium* parasites was from *Theileria parva*. This was to be expected since *T. parva* belongs to the same phylum as *Plasmodium* parasites, the Apicomplexans [31]. Closer analysis of the *Plasmodium* sequences indicated that the three rodent-infecting malaria parasites cluster together, whilst *P. vivax*, *P. knowlesi* and *P. falciparum* form a separate but related cluster. Similar relationships have been observed from a phylogenetic analysis of the *Plasmodium* cytochrome-b and small subunit rRNA sequences [32]. The separate cluster of the mammalian, yeast and plant reference sequences suggests a more distant similarity to the *Plasmodium* sequences.

Considering its clinical importance, *P. falciparum* was selected for further studies of the putative copper transporter. The gene encoding the putative copper transport protein (PF14_0369) is located on the positive strand of chromosome 14 as a two-exon gene with a 336 bp first exon and 372 bp second exon, translating to a 235 amino acid protein with a predicted mass of 27.15 kDa. The full length and amino terminal coding domain coding sequences for the putative *P. falciparum* copper transport protein (PF14_0369) were PCR amplified from genomic DNA, producing amplicons of approximately 890 bp and 290 bp, respectively (Figure 1D). An 820 bp product was amplified for the LDH control (Figure 1D). The identity of the amplicon for the putative transporter’s amino terminal domain (*Pf*Ctr369Nt^-S^) was confirmed by sequencing. This amplicon was cloned and expressed as a MBP fusion partner (MBP-*Pf*Ctr369Nt^-S^).

### Recombinant expression of MBP-*Pf*Ctr369Nt^-S^

**Figure 2:**
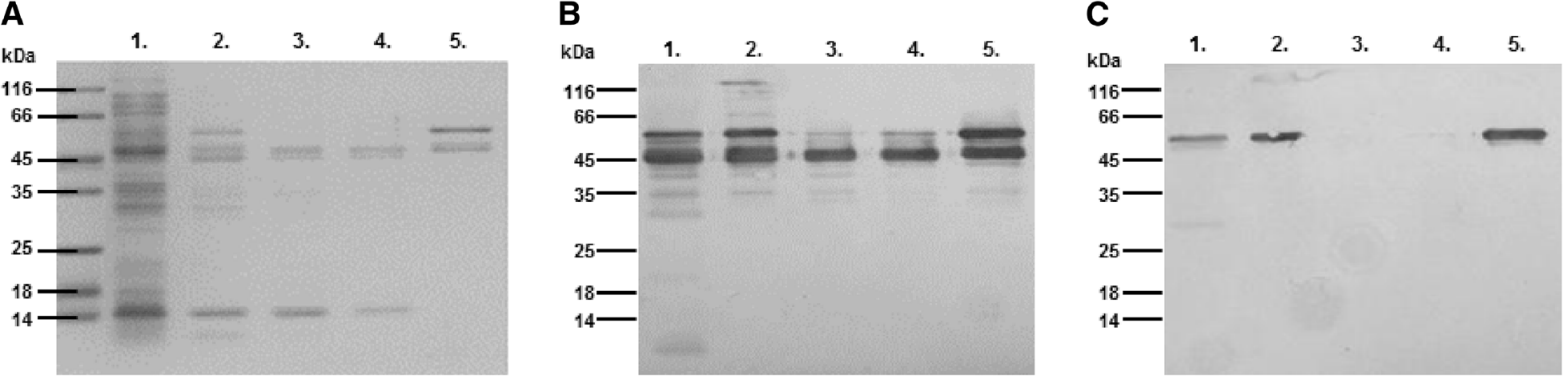
**Expression and purification of recombinant MBP-*Pf*Ctr369Nt**^**-S**^**.** Expression of MBP-*Pf*Ctr369Nt^-S^ was targeted to the *E. coli* periplasm. **A**. Steps in the isolation of recombinant MBP-*Pf*Ctr369Nt^-S^ were analysed on a 12.5% reducing SDS-PAGE. Western blot of A. probed with either anti-MBP **B**. or anti-PF14_0369 antibodies **C**. In all panels: lane 1, total *E. coli* lysate; lane 2, periplasmic proteins; lanes 3–5 represent proteins that did not bind, were washed from and eluted off the amylose resin, respectively. Molecular markers are shown to the left of each image.

The amino terminal domain of yeast and mammalian copper transport proteins is essential for copper acquisition [33], hence the corresponding domain of the putative *P. falciparum* copper transporter was selected for copper binding studies. A similar approach has been adopted for characterization of the *Pf*CuP-ATPase copper efflux pump [5]. To limit the possibility of recombinant MBP-*Pf*Ctr369Nt^-S^ associating with a bacterial membrane, the predicted signal peptide was omitted and denoted as “-S” [34]. Expression of a 55 kDa protein was induced from the MBP-*Pf*Ctr369Nt^-S^ plasmid and affinity purified from the bacterial periplasmic contents using an amylose affinity matrix (Figure 2A). The size of the purified protein corresponded well with that predicted from the cloned sequence (54 kDa). An additional 42 kDa product, in the purified MBP-*Pf*Ctr369Nt^-S^ fraction, is likely to be MBP lacking the fused protein. A Western blot using anti-MBP antibodies recognizes both the 42 kDa and 55 kDa bands (Figure 2B), whilst antibodies specific for *Pf*Ctr369Nt^-S^ only recognize the 55 kDa band (Figure 2C). Also evident in the anti-MBP (Figure 2B) and anti-*Pf*Ctr369Nt^-S^ blots (Figure 2C) are additional truncated forms of MBP-*Pf*Ctr369Nt^-S^ between the 55 kDa and 42 kDa bands. Recombinant MBP-*Pf*Ctr211Nt^-S^ was expressed as a 46 kDa protein (Additional file 2).

### MBP-*Pf*Ctr369Nt^-S^ binds copper

**Figure 3:**
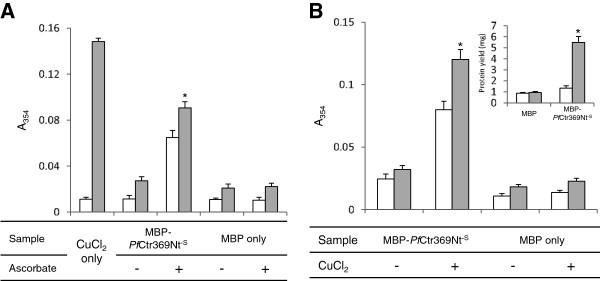
**MBP-*Pf*Ctr369Nt**^**-S**^**binds copper *in vitro *and within *E. coli *host cells.A**. Affinity-purified MBP-*Pf*Ctr369Nt^-S^ or MBP was incubated with CuCl_2_ in the presence (+) or absence (−) of ascorbic acid *in vitro*. The BCA release assay detected copper with (solid bars) or without (open bars) the addition of ascorbic acid. The concentration of the copper standard (CuCl_2_ only) was equimolar to the amount of protein used. **B**. CuCl_2_ was added to the *E. coli* cell growth medium after the induction of recombinant protein expression. Following affinity purification, protein-bound copper was detected using the BCA release assay with (solid bars) or without (open bars) the addition of ascorbic acid. The addition of copper to the *E. coli* growth medium increased recombinant protein yield (inset). In all panels, MBP serves as a control and * represents statistical significance (p < 0.05). Results are means ± S.D. of triplicate measurements done for duplicate dialysis sacs.

In yeast and mammalian cells, copper appears to be transported across the membrane in its reduced form (Cu^+^) [30] and hence a Cu^+^ preference was examined for the putative *P. falciparum* copper transporter. Purified MBP-*Pf*Ctr369Nt^-S^ was incubated with CuCl_2_ in the presence or absence of the reducing agent ascorbate. Unbound copper was removed by dialysis and the bound copper detected using the BCA assay [26] with or without a second treatment of ascorbate. If ascorbate was required in the BCA assay for the formation of a purple complex it suggests Cu^2+^ is preferentially bound. Alternatively, if ascorbate supplementation was not required for colour formation then Cu^+^ is suggested to be the preferred substrate. Purified MBP-*Pf*Ctr369Nt^-S^ showed a binding preference for Cu^+^*in vitro* (Figure 3A). Exclusion of ascorbate from the initial CuCl_2_ incubation resulted in low levels of detectable copper (Figure 3A), whereas including ascorbate resulted in a ~70% increase (p < 0.01) in the amount of copper detected. The absence of ascorbate from the BCA assay did not affect BCA-Cu^+^ complex formation for the sample originally incubated with ascorbate, thereby supporting a preference for Cu^+^ (Figure 3A).

The ability of MBP-*Pf*Ctr369Nt^-S^ to bind copper within a cellular environment was examined by expressing the protein in the presence of 0.5 mM CuCl_2_. This concentration of copper does not affect *E. coli* growth [35]. MBP-*Pf*Ctr369Nt^-S^ isolated and affinity purified from *E. coli* cells grown in copper-enriched media, bound copper (Figure 3B). Extraneous copper was removed by washing the cells prior to protein isolation. Protein isolated from cells grown without copper had negligible amounts of copper present (p < 0.01) (Figure 3B). The oxidation state of copper bound within the cell was inferred by including or excluding ascorbate from the BCA assay (Figure 3B). Like the *in vitro* analysis, Cu^+^ appeared to be the preferred oxidation state bound by the protein within the *E. coli* periplasm. An interesting consequence when copper was added to the *E. coli* growth medium was a four-fold increase in MBP-*Pf*Ctr369Nt^-S^ yield from 1.53 mg to 5.98 mg per litre of culture (p < 0.01) (Figure 3B, inset). In all experiments MBP served as a negative control. Recombinant MBP did not bind copper and CuCl_2_ had no perceptible influence on MBP expression (Figure 3).

### Analysis of copper binding to MBP-*Pf*Ctr369Nt^-S^ using the rate of ascorbate oxidation

**Figure 4:**
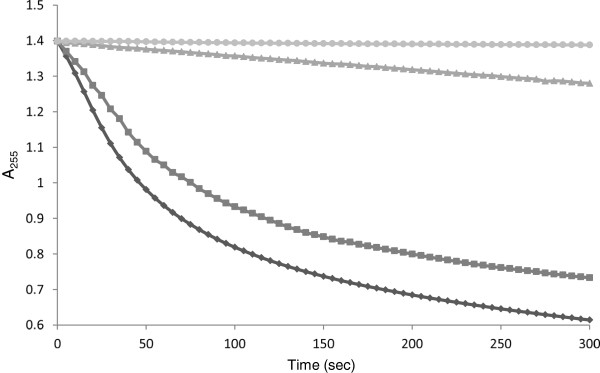
**MBP-*Pf*Ctr369Nt**^**-S**^**inhibits the** c**opper-catalysed oxidative degradation of ascorbate.** Ascorbate does not auto-oxidize when exposed to air (indicated by the light gray circle). The addition of CuCl_2_ catalyses the oxidation of the ascorbate solution (indicated by the dark gray diamond), with this oxidation rate reduced by the inclusion of MBP-*Pf*Ctr369Nt^-S^ (indicated by the light gray triangle). MBP (indicated by the dark gray square) did not prevent the copper-catalysed oxidation of ascorbate.

By taking advantage of the copper-catalysed oxidation rate of ascorbate and the ability of chelators to affect this rate, Jiang *et al.*[36] demonstrated that copper transport protein methionine motif peptides bind Cu^+^ with methionine-only coordination. The effect MBP-*Pf*Ctr369Nt^-S^ had on copper-catalysed ascorbate oxidation was examined by monitoring the loss of absorbance at 255 nm of a 120 μM ascorbate solution at pH 4.5. This pH was optimal for several reasons. First, increased concentrations of the ascorbate monoanion (HAsc^-^), which has a pKa of 4.1, cause increased oxidation rates thus elevated pH values can amplify unwanted errors. Second, Cu^2+^ hydrolysis is minimal at a lower pH [36]. Last, and perhaps more relevant to the amino terminal domain of the putative *P. falciparum* copper transporter, is that the related human Ctr1 shows increased copper uptake at a low extracellular pH and yeast cells expressing Ctr1 acidify their media to pH 4 – 5 [37]. Freshly prepared ascorbate showed a stable absorbance at 255 nm (A_255_) over 300 seconds (Figure 4). The addition of 8 μM CuCl_2_ caused a rapid decrease in the A_255_ signal, indicative of oxidation by copper. The copper-catalysed oxidation appeared to be inhibited by the addition of 5 μM purified MBP-*Pf*Ctr369Nt^-S^ (Figure 4). This suggested MBP-*Pf*Ctr369Nt^-S^ chelates copper in solution, thus preventing its participation in the redox cycle of ascorbate oxidation. Although this assay is not definitive of the copper species preferentially bound by MBP-*Pf*Ctr369Nt^-S^, it is presumably Cu^+^, based on results from the other copper-binding studies conducted for MBP-*Pf*Ctr369Nt^-S^ (Figure 3). Control experiments indicated MBP had minimal influence on the copper-catalysed oxidation rate of ascorbate.

### Native expression of a putative *P. falciparum* copper transport protein

**Figure 5:**
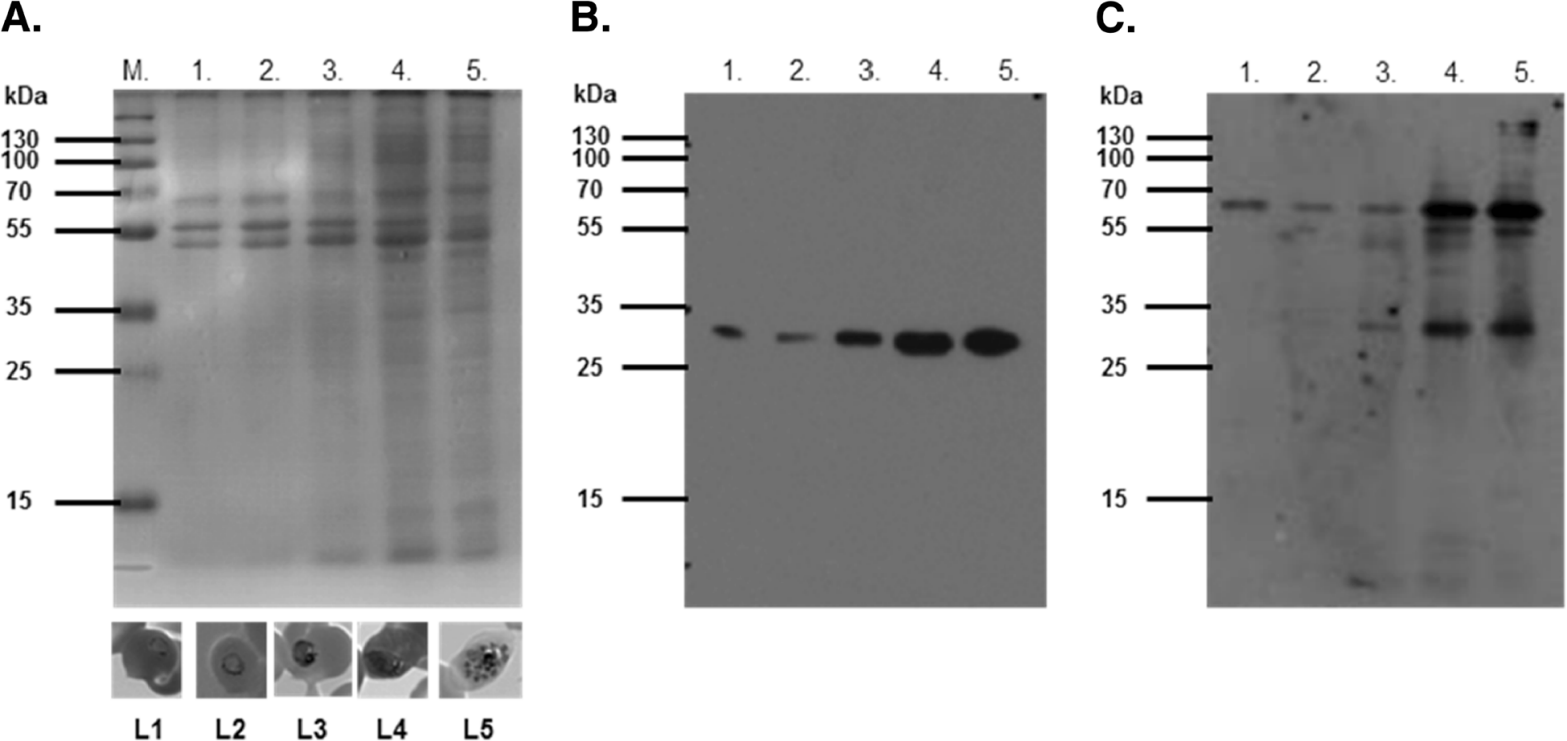
**Expression of the putative*P. falciparum*copper transporter (PF14_0369) in synchronized asexual parasites.** Asexual *P. falciparum* parasites were grown *in vitro*, synchronized by sorbitol treatment and samples removed at five time points. Equivalent amounts of 1 x 10^7^ synchronous, saponin-isolated *P. falciparum* parasites were analysed by **A**. 12.5% reducing SDS-PAGE, blotted onto nitrocellulose and probed with chicken anti-LDH peptide antibodies **B**. and anti-PF14_0369 peptide antibodies **C.** In all panels: lane 1 (L1), early rings; lane 2 (L2), late rings/early trophozoites; lane 3 (L3), trophozoites; lane 4 (L4), late trophozoites; lane 5 (L5), schizonts. Representative images of each stage of asexual development are shown below panel A (L1 – L5). M are the prestained molecular marker proteins.

An antibody probe specific for the putative *P. falciparum* copper transporter (PF14_0369) was designed to detect the native *P. falciparum* protein. Parasite lysates from sorbitol-synchronized *P. falciparum* parasite cultures were separated by SDS-PAGE (Figure 5A) and transferred to a nitrocellulose membrane. Affinity purified anti-peptide antibodies targeting the N-terminal domain of the putative *P. falciparum* copper transporter recognized protein bands of 34 kDa and 68 kDa in most stages of sorbitol-synchronized, asexual cultured parasites (Figure 5C). These bands may represent monomeric and dimeric species, as has been suggested for human Ctr1 [37]. Expression of the putative copper transporter appeared to increase in a similar manner to *Pf.* LDH as *P. falciparum* progressed through its asexual cycle (Figure 5B). The increase in protein expression is also seen as an increase in PF14_0369 transcription [38].

### Localization of a putative *P. falciparum* copper transport protein

Characterized copper transport proteins are defined as integral membrane proteins that, depending on cell type, localize to either the plasma membrane or intracellular vesicles [8]. Analysis of the PF14_0369 protein sequence predicted an amino terminal signal peptide [39] and an apicoplast targeting signal [40]. Further analysis indicated that PF14_0369 lacked an identifiable PEXEL/HT motif. This suggested the putative copper transporter would be targeted to the parasite’s apicoplast and not to the parasite or erythrocyte plasma membranes. To define the protein’s native sub-cellular location, the anti-peptide antibodies specific for the putative copper transporter were used in immunofluorescence assays. For this assay a modified form of the fixation protocol developed by Tonkin *et al.*[27] was employed. Advantages of this technique, over the traditional methanol or methanol/acetone fixation methods, include decreased auto-fluorescence and improved preservation of cell morphology [27].

**Figure 6:**
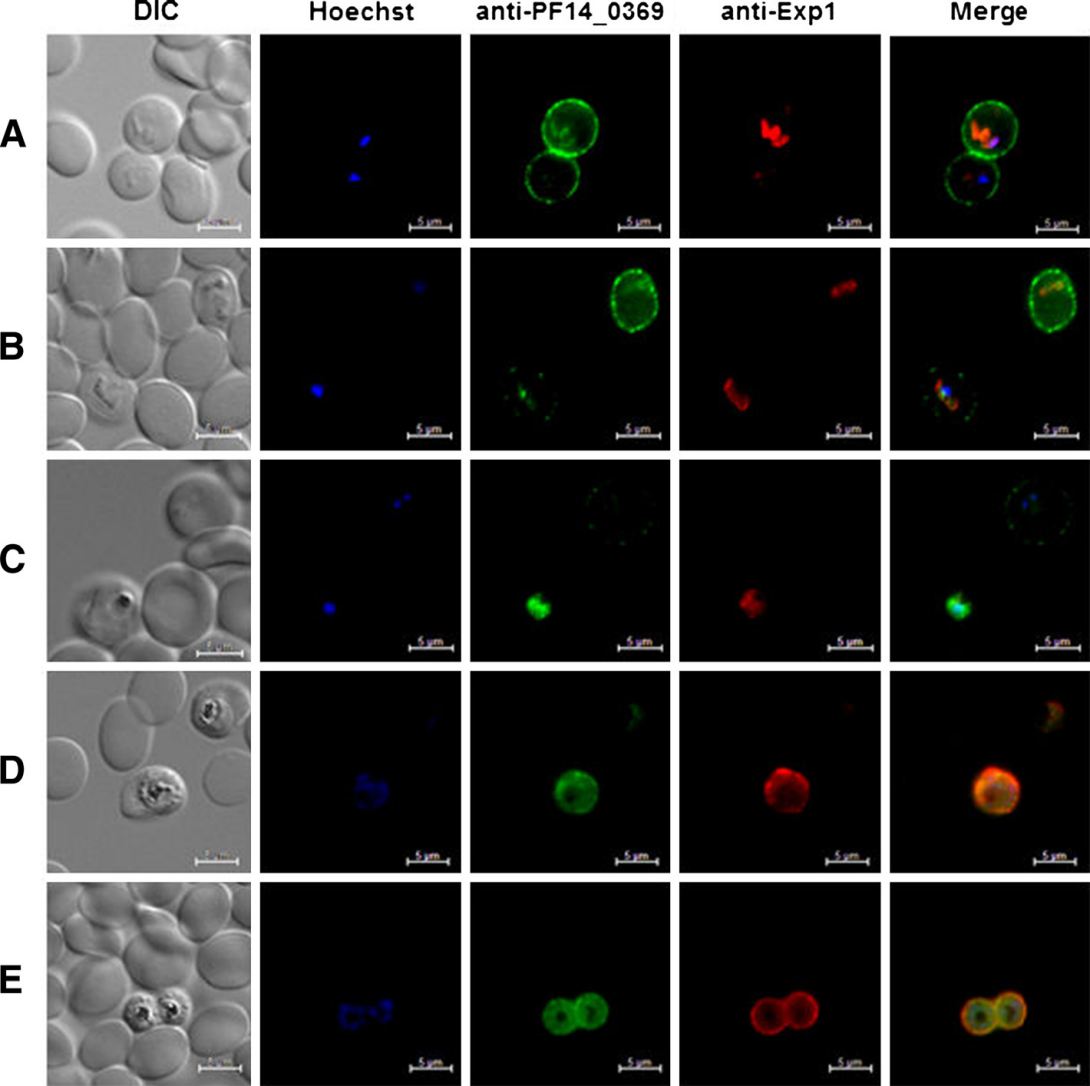
**Immunolocalization of a putative *P. falciparum *copper transport protein.** Asexual *P. falciparum* parasites were grown *in vitro*, synchronized by sorbitol treatment and samples removed at five time points (**A** – **E**). Fixed parasites were probed with chicken anti-peptide antibodies targeting PF14_0369 and rabbit antibodies against Exp-1. Chicken antibodies were detected with a Cy3-conjugated antibody and rabbit antibodies with a Cy2-conjugated antibody. Panels are representative of the following stages of asexual parasite development: **A**. early rings; **B**. late rings; **C**. early trophozoites; **D**. late trophozoites; **E**. schizonts. Parasite DNA was stained with Hoechst. Scale bar is 5 μm.

Sorbitol-synchronized parasites at various developmental stages were fixed as described above and probed with specific anti-peptide antibodies. In ring stage parasites, the majority of the fluorescence signal could be detected in close proximity to the erythrocyte plasma membrane (Figure 6A), with lower amounts of labelling found close to the body of the parasite. The signal associated with the parasite body overlaps considerably with that for Exp-1, a marker of the parasitophorous vacuole membrane [41]. As parasites progressed through the erythrocytic cycle, the erythrocyte plasma membrane staining became progressively weaker, with a concomitant increase in the signal co-localizing with Exp-1 (Figure 6B-E). The specificity of this signal migration becomes even more apparent in panels in which two different parasites of slightly different developmental progression show differing fluorescence phenotypes (Figure 6B and C). Towards the later stages of parasite development, the entire fluorescence signal can be seen in tight association with the parasite itself, with no signal detectable in the erythrocyte (Figure 6D and E). As the copper transporter is predicted to contain apicoplast targeting information, we carried out immuno-colocalization studies using antisera detecting the apicoplast resident protein acyl-carrier protein. Even in parasite stages in which the copper transporter appears to localize with the body of the parasite, no significant signal co-localization was noted. All images were acquired using the same exposure time to allow for direct comparison of signal strength. Control experiments using pre-immune antibody preparations or only secondary antibodies showed no antibody staining and additionally experiments targeting the *P. falciparum* cytoplasmic proteins LDH, GAPDH and HSP86/90 showed a notably different localization from that of the putative copper transporter. No signal was seen to associate with non-infected erythrocytes (Figure 6A-E).

### Analysis of PF14_0369 for novel export signals

Export of the putative copper transporter to the erythrocyte surface was surprising considering the lack of an identifiable PEXEL/HT motif. Exported *P. falciparum* proteins lacking a PEXEL motif have previously been identified and are termed “PEXEL negative exported proteins” (PNEPs) [42]. Partial PEXEL motifs have been identified in the amino terminal domain of the PNEPs REX2 (LAE) [43] and SBP1 (LAD) [44] that are essential for export. Inspection of the PF14_0369 amino terminal domain identified a partial PEXEL motif identical to the important LAD motif identified in SBP1. Analysis of the essential transmembrane domains from SBP1 and REX2 highlighted a relative abundance of phenylalanine residues [43] also found in the third transmembrane domain of PF14_0369 (Additional file 1).

## Discussion

Maintenance of copper homeostasis is essential for cell survival. Consequently sophisticated mechanisms have evolved for copper acquisition, distribution and excretion. In mammalian cells, copper is acquired by the copper transport protein Ctr1 and distributed to target proteins via dedicated copper metallochaperones such as CCS, Atox1 and Cox17 [7,8]. To avoid the potentially toxic effects of copper, Cu-ATPase proteins mediate the excretion of excess copper [9] and a *P. falciparum* Cu-ATPase ortholog has been identified [5]. One *P. falciparum* copper source appears to be from the ingestion of host erythrocyte Cu/Zn SOD [5]. A screen of the PlasmoDB database, performed in the present study, identified six *P. falciparum* copper-requiring protein orthologs and a candidate copper transport protein. Native expression of the putative copper transporter was confirmed by Western blot and the protein’s subcellular location identified by immunofluorescence microscopy. A recombinant form of the transporter's amino terminal domain was shown to bind copper *in vitro* and within *E. coli* host cells, supporting the possibility that the full-length protein functions to acquire copper.

### Identification of a putative *P. falciparum* copper transport protein

The identification of six *P. falciparum* copper-requiring protein orthologs suggested an important role for copper in *P. falciparum* metabolism. Treating *P. falciparum* parasites with the intracellular copper chelator, neocuproine, inhibited ring-to-trophozoite transition *in vitro*[5], highlighting the importance of copper for the parasite. During the course of its asexual cycle, *P. falciparum* appears to digest host erythrocyte Cu/Zn SOD to release copper [5]. The presence of a membrane associated copper binding protein with copper transport protein characteristics in plasmodia suggests a mechanism for the transport of copper similar to that played by the Ctr1 copper transport protein in yeast and mammalian cells [7,8]. Candidate copper transporter sequences were identified for eight species of the *Plasmodium* parasite and each sequence was shown to contain the essential and largely definitive Mx_3_M and Gx_3_G motifs (Figure 1B) [30,45]. The predicted amino terminal domain of each putative transporter was found to contain one or more methionine motifs (MxM or MxxM). Although not limited to copper transporters, these motifs are important for Ctr1 protein function [30].

The presence of three putative membrane-spanning regions is considered definitive for copper transport proteins [33] and topological analysis of the putative *P. falciparum* copper transporter identified three such domains. Since three transmembrane domains cannot form a functional channel or pore for ion transport [10], a homotrimeric complex of copper transport protein monomers is formed for the characterized mammalian and yeast copper transport proteins [33]. The *P. falciparum* copper transport protein may form a similar complex since the first transmembrane domain of the copper transport protein family serves as an adaptor allowing evolutionarily distant copper transporters to adopt a similar overall structure [46]. An immunoblot of yeast and mammalian copper transport proteins identified monomeric and dimeric species of the trimeric complex [37]. Similar monomeric and dimeric species were detected in an immunoblot of the putative *P. falciparum* copper transport protein in this study.

### Recombinant expression and copper binding to MBP-*Pf*Ctr369Nt^-S^

Signal peptides, transmembrane domains, rare codons, introns and genome AT-richness affect recombinant expression of *P. falciparum* proteins [34]. The amino terminal signal peptide of the putative *P. falciparum* copper transporter was, therefore, excluded from the expression construct, resulting in periplasmic expression. The purified MBP-*Pf*Ctr369Nt^-S^ recombinant protein bound reduced copper *in vitro* and within a cellular environment. A second copper transport protein, MBP-*Pf*Ctr211Nt^-S^, from the PF14_0211 gene was recombinantly expressed and isolated. Expression of the native *P. falciparum* protein encoded by this gene has not been ascertained at this stage.

In mammalian and yeast cells, the reduced cuprous ion (Cu^+^) is favoured for transfer and transport [30], since Cu^+^ is more exchange labile than Cu^2+^[36]. Cu^+^ is highly reactive in an oxidizing environment and methionine motifs in the extracellular amino terminal domain of the copper transporter family are thought to sequester copper prior to its transport across the lipid bilayer [30]. This was supported by a study demonstrating that methionine motif peptides inhibited copper-catalysed ascorbate oxidation through Cu^+^ chelation [36]. MBP-*Pf*Ctr369Nt^-S^ inhibited copper-catalysed ascorbate oxidation through copper chelation. Taken together with the data showing copper binding to MBP-*Pf*Ctr369Nt^-S^, it appeared that the amino terminal domain of the putative *P. falciparum* copper transporter preferably coordinates the Cu^+^ ion. Copper coordination was presumably a result of the methionine motif present in MBP-*Pf*Ctr369Nt^-S^.

The methionine motifs contained in the amino terminal domains of yeast and human copper transport proteins are essential for copper binding under copper limiting conditions [30]. The yeast copper transport protein contains eight methionine motifs, but only the last methionine of the eighth motif was shown by Puig *et al.*[30] to be essential for copper binding capability. The position of this methionine is conserved between copper transport proteins and is 20 amino acid residues N-terminal to the transporter’s first transmembrane domain [30]. Interestingly, the last methionine of the methionine motif in the amino terminal domain of the putative *P. falciparum* copper transport protein is located in a similar position. However, the involvement of other amino acid residues, like cysteine and histidine residues that bind metal ions [47,48], and are present in this protein cannot be excluded. Identification of the specific residues coordinating copper will be explored in future experiments.

### MBP-*Pf*Ctr369Nt^-S^ recombinant protein likely interacts with *E. coli* copper binding proteins

An interesting consequence of expressing MBP-*Pf*Ctr369Nt^-S^ in the presence of copper was a significant increase in recombinant protein yield. In the presence of excess copper it is a possibility that the copper bound to and stabilized the recombinant protein [48]. The cytoplasm and periplasm of *E. coli* cells do, however, have different copper requirements since almost all bacterial copper proteins, such as multi-copper oxidases, amine oxidases and lysine oxidases are found in the periplasm or excreted extracellularly [49]. Copper availability in *E. coli* is thought to be regulated by the actions of the DNA-binding metal sensor CueR, which controls the expression of genes encoding proteins involved in metal homeostasis [50]. A copper-requiring protein is thought to gain access to copper only if the protein’s affinity for the metal ion is greater than the buffered cellular concentration of copper [51]. Interaction of a recombinant *P. falciparum* protein with native *E. coli* proteins has been demonstrated for *Pf*Hsp70 [52], supporting the possibility that MBP-*Pf*Ctr369Nt^-S^ interacts with native *E. coli* copper proteins for copper loading. These interactions could, in turn, influence the availability of copper for native *E. coli* copper-requiring proteins resulting in tightly regulated MBP-*Pf*Ctr369Nt^-S^ expression under standard growth conditions. The addition of excess extracellular copper appears to have alleviated this growth stress producing the increase in the expression of MBP-*Pf*Ctr369Nt^-S^ observed here.

### The putative *P. falciparum* copper transport protein shows stage-specific localization

During early stages of asexual development, the putative *P. falciparum* copper transporter appears to be targeted to the plasma membrane of the infected erythrocyte. As the parasite matures through its asexual cycle the protein was detected on a parasite membrane. This may represent a turnover of host cell membrane associated malarial proteins, followed by trafficking of the copper transporter to a parasite membrane. The precise localization of the copper transporter, when associated with the parasite, was not elucidated, due to limits to the fluorescence detection methods. The parasitophorous vacuolar membrane contains a “promiscuous” pore that permits the passage of solutes, nutrients and macromolecules [53,54] and thus it is unlikely for there to be the need for a separate copper transporter. The parasite plasma membrane contains a variety of selective transporters (Reviewed by Martin *et al.*[4]). The copper transporter is thus suggested to be associated with the parasite plasma membrane. *Pf*CuP-ATPase was similarly localized to a parasite plasma membrane and the surface of the infected erythrocyte. However, unlike the putative copper transport protein, *Pf*CuP-ATPase was associated with both the parasite plasma membrane and the erythrocyte membrane at the same time in trophozoites and schizonts, whilst no expression was detected in rings [5]. These authors suggested that dual localization of *Pf*CuP-ATPase represents a novel mechanism by which the parasite reduces copper toxicity through copper efflux [5]. Reasons for the different localities of the copper transport protein are less apparent. There is the possibility that during ring stages, extracellular copper is required until the parasite digests endocytosed host cell Cu/Zn SOD and translocates the copper transporter protein to a parasite membrane.

The concentration of copper in normal red blood cells has been reported as18 μM and copper is imported into red cells by a Cl^-^-HCO_3_^-^ anion exchanger [55]. Inside the red cell most of the copper is associated with Cu/Zn SOD and a 30 and a 40 kDa red cell protein [56]. Rasoloson *et al.*[5] reported a lower red cell copper concentration at 10 μM and further suggested that copper concentrations in parasitized cells was lower than uninfected cells. Copper in isolated parasites was lower than in red cells at 2 μM in rings and between 6 and 6.6 μM in trophozoites (depending on the isolation method). This data disagrees with recent data indicating that malaria infected red cell copper concentration was 30 μM, and double that of uninfected red cells in the same experiment [57]. The different values for copper concentrations in infected red cells may be due to the different measuring methods employed and needs to be reconciled [5,57]. Interestingly as the parasite develops from trophozoites to schizonts, the concentration of ingested Cu/Zn SOD decreases [5] and this coincides with data presented here showing the appearance of the putative copper transporter on parasite membranes, the highest level of expression of the protein, and mRNA levels described by Le Roch *et al.*[38]. Thus as the source of copper in the form of Cu/Zn SOD decreases in the parasite [5], the parasite compensates with an increase in expression of the putative copper transport protein. Given the potential toxicity of copper and the need for the regulation of copper homeostasis, it is likely that the parasite employs a copper transport protein alongside the *Pf*CuP-ATPase copper export protein described by Rasoloson *et al.*[5]. It is suggested that, given the presence and membrane location of a copper binding protein with copper transporting motifs, and the increase in copper concentration in malaria infected red cells [57], that the protein described here has a role in copper transport. Data to support a transport role for the copper binding protein is being pursued.

Treatment of *P. falciparum* parasites with neocuproine inhibited ring-to-trophozoite transition [5], implicating copper metabolism as a potential target for novel anti-malarial drug development. Copper, when the concentration is above a critical level, has been shown to be toxic to the parasite [58]. However, from *in silico* findings it seems unlikely that *P. falciparum* contains unique copper-dependent metabolic pathways. Copper-binding protein motifs also appear to be conserved from prokaryotes to eukaryotes [59], suggesting that designing a compound specific for *P. falciparum* copper-dependent proteins could prove difficult. An alternative approach would be to deliver anti-malarial compounds to the parasite by exploiting transport mechanisms, as suggested for the *P. falciparum* new permeation pathway and choline carrier [60]. The human copper transport protein has been shown to transport the platinum containing anti-cancer drugs cisplatin and oxaliplatin [61]. Cisplatin also exhibits anti-malarial activity through DNA damage [62,63]. Given the structural similarities between the putative *P. falciparum* copper transport protein and the human copper transporter, it is possible that cisplatin is delivered to the parasite by the putative copper transport protein. This transport mechanism could perhaps be exploited for the delivery of novel platinum-based anti-malarial compounds.

*Plasmodium falciparum* actively remodels the erythrocyte during infection, leading to an increase in the permeability of the host cell membrane to low molecular weight solutes [64]. This increase is mediated by ‘new permeability pathways’ that have also been shown to greatly facilitate the uptake of the antibiotics fosmidomycin and its derivative FR900098 [62]. Parasitized erythrocytes also show a significant increase in the uptake of a copper-neocuproine complex when compared to uninfected erythrocytes [65]. Association of the putative *P. falciparum* copper transport protein with the erythrocyte membrane, during early asexual development, therefore makes it tempting to speculate that the copper transport protein’s mechanism accounts for the increased rate of copper-neocuproine complex uptake by parasitized erythrocytes. Complexation of the anti-malarial buparvaquone to copper(II) significantly enhanced its anti-malarial activity [66]. This was proposed to be a consequence of improved compound internalisation, which may be related to the transport mechanism of the putative copper transport protein. Localization of the *P. falciparum* copper transport protein to the erythrocyte and parasite membranes in late ring and early trophozoite stages may explain the increased susceptibility of these stages to cisplatin [67].

### The putative copper transport protein sequence lacks an identifiable export motif

*Plasmodium falciparum* protein export beyond the parasitophorous vacuole membrane was suggested to be mediated by the PEXEL/HT motif, but this motif was later shown not to be the sole determinant of protein export. Over 300 PEXEL/HT proteins have been predicted, whereas only a few PEXEL negative export proteins (PNEPs) have been identified and a common PNEP motif is yet to be identified [68]. One important feature of PNEP proteins is the presence of a transmembrane domain, since removal of this domain from the PNEPs MAHRP1, SBP1 and REX2 inhibited protein export [43,44]. Similarly, the transmembrane domain of SURFIN_4.2_ was essential for protein trafficking to the infected erythrocyte and Maurer’s clefts [69].

Analysis of the PNEPs REX2 [43] and SBP1 [44] identified partial PEXEL/HT motifs (LAE and LAD, respectively) as being essential for protein export. Analysis of the PF14_0369 sequence identified an identical LAD motif within the first 20 amino acids of its amino terminal domain, a similar position to the LAD motif in SBP1. Mutational analysis of the related LAE motif in REX2 established that the glutamate residue was essential for export [43]. This suggests the related aspartate residue in the LAD motif of PF14_0369 may be important for export. Cleavage of the signal peptide in the PF14_0369 sequence would generate a new amino terminus similar to that generated following cleavage of the PEXEL motif in PEXEL/HT proteins. PEXEL motif cleavage generates an xD/E/Q amino terminus, with the presence of D/E/Q being essential for protein export [70]. Another potential export signal in SBP1 is located N-terminal of its transmembrane domain, between amino acids 180 and 190 [44]. A comparison of this short signal sequence (NEYEVES) with the PF14_0369 sequence identified a similar sequence upstream of the first predicted transmembrane domain (NKWETKS), thereby implicating this sequence as a potential contributor to successful protein export. The presence of motifs in PF14_0369 similar to those important for PNEP export suggests that these motifs play a role in the export of the putative copper transporter.

The amino terminal domain of the *P. falciparum* copper binding protein described here binds copper *in vitro* and in an *in vivo* expression system. The protein has copper transport motifs and has been shown to be expressed by malaria parasites and locate to two different membranes (the erythrocyte and the parasite membrane) as the parasite develops within the infected red cell. This evidence implicates a copper transport role for the protein in malaria infected erythrocytes and this implication is being explored. Whether the protein imports copper or has a copper export role alongside the Cu-ATPase protein described by others [5], remains to be elucidated.

## Conclusions

A putative *P. falciparum* copper transport protein was identified and showed that the recombinant amino terminal domain binds copper *in vitro* and within *E. coli* host cells. This ability, combined with conserved copper transport protein sequence features, suggest an additional malaria copper transport mechanism to that previously described [5]. Interestingly, the putative *P. falciparum* copper transport protein initially localized to the erythrocyte membrane and then relocated to a parasite membrane through the course of asexual development. The presence of a putative malaria copper transport protein has interesting implications for malaria copper metabolism and the protein may have a potential role as a novel anti-malarial drug delivery system.

## Abbreviations

Atox1: Antioxidant protein 1; BCA: Bicinchoninic acid; CCO: Cytochrome-c oxidase; CCS: Copper chaperone for superoxide dismutase; Ctr1: Copper transport protein 1; Cu/Zn SOD: Cuprozinc superoxide dismutase; Exp1: Export protein 1; GAPDH: Glyceraldehyde 3-phosphate dehydrogenase; H_2_ Asc: Ascorbic acid; HSP 86/90: Heat shock protein 86/90; LDH: Lactate dehydrogenase; MAHRP1: Membrane-associated histidine-rich protein 1; MBP: Maltose binding protein; PEXEL/HT: *Plasmodium* export element/host targeting signal; *Pf*Ctr369Nt^-S^: *Plasmodium falciparum* copper transport protein PF14_0369 amino terminal domain minus signal peptide; PNEP: PEXEL negative exported protein; REX2: Ring exported protein 2; SBP1: Skeleton binding protein 1.

## Competing interests

The authors declare that they have no competing interests.

## Authors’ contributions

All authors contributed to planning the experimental strategy, analysing the data and writing the paper. DLC conducted the experiments with the guidance of JPDG. DLC conducted the immunofluorescence experiments with help from JMP. All authors read and approved the final manuscript.

## Supplementary Material

Additional file 1 **Important features of the PF14_0369 amino acid sequence.** Important features of the PF14_0369 amino acid sequence include a predicted N-terminal signal peptide (underlined), three transmembrane domains (black boxes and numbered 1,2,3), an essential methionine residue **M**, 20 amino acids N-terminal of the first transmembrane domain, and the MX_3_M and GX_3_G motifs. Features thought to contribute to protein trafficking include a partial PEXEL motif (**LAD**) in the signal peptide and an enrichment of phenylalanine residues (**F)** in the third transmembrane domain. Click here for file

Additional file 2 **Expression and purification of recombinant MBP-*Pf*Ctr211Nt**^**-S**^**.** Expression of MBP-*Pf*Ctr211Nt^-S^ was targeted to the *E. coli* periplasm. Steps in the isolation of recombinant MBP-*Pf*Ctr211Nt^-S^ were analysed on a 10% reducing SDS-PAGE. Lane 1 and 2, total *E. coli* lysate; lane 3, periplasmic proteins; lane 4 represents proteins that did not bind and lanes 5–10 show protein eluted off the amylose resin. Fermentas unstained protein marker standards are shown to the left of each image. Click here for file
