# Supplementary figures and images for: A Plasmodium falciparum copper-binding membrane protein with copper transport motifs
Source: Malar J. 2012 Nov 29;11:397. doi: 10.1186/1475-2875-11-397 (PMC3528452; doi:10.1186/1475-2875-11-397)

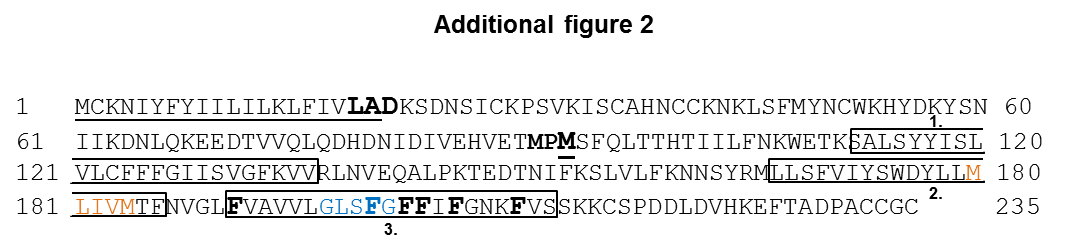

Supplement: Additional file 1 — Important features of the PF14_0369 amino acid sequence. Important features of the PF14_0369 amino acid sequence include a predicted N-terminal signal peptide (underlined), three transmembrane domains (black boxes and numbered 1,2,3), an essential methionine residue M, 20 amino acids N-terminal of the first transmembrane domain, and the MX3M and GX3G motifs. Features thought to contribute to protein trafficking include a partial PEXEL motif (LAD) in the signal peptide and an enrichment of phenylalanine residues (F) in the third transmembrane domain. [file 1475-2875-11-397-S1.png]

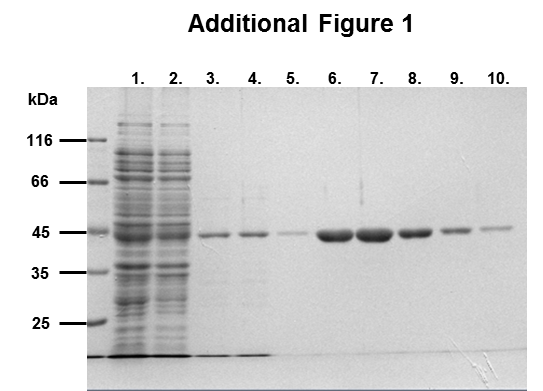

Supplement: Additional file 2 — Expression and purification of recombinant MBP-PfCtr211Nt-S. Expression of MBP-PfCtr211Nt-S was targeted to the E. coli periplasm. Steps in the isolation of recombinant MBP-PfCtr211Nt-S were analysed on a 10% reducing SDS-PAGE. Lane 1 and 2, total E. coli lysate; lane 3, periplasmic proteins; lane 4 represents proteins that did not bind and lanes 5–10 show protein eluted off the amylose resin. Fermentas unstained protein marker standards are shown to the left of each image. [file 1475-2875-11-397-S2.png]
